# Evaluation of Physician’s Attitudes and Knowledge Regarding the Diagnosis of Brain Death in Deceased Organ Transplantation in Northern Cyprus

**DOI:** 10.7759/cureus.40749

**Published:** 2023-06-21

**Authors:** Necmi Bayraktar, Omer Tasargol

**Affiliations:** 1 Urology, Dr. Burhan Nalbantoglu State Hospital, Nicosia, CYP; 2 Anesthesiology, Dr. Burhan Nalbantoglu State Hospital, Nicosia, CYP

**Keywords:** brain death diagnosis, organ transplantation, organ donation rates, overcome organ shortage, deceased organ transplantation rate

## Abstract

Introduction

Increasing deceased organ transplantation rates is an important strategy to overcome the organ shortage. Prior to the pandemic in Northern Cyprus, there were more transplants from deceased donors than from living donors. However, after the pandemic, living donor organ transplants were almost equal to cadaveric organ transplants. The purpose of this study was to explore the knowledge, attitudes, and experiences of hospital-based clinicians involved in the diagnosis of brain death and donor care in order to raise the deceased organ transplantation rate.

Methods

The study population consisted of three departments: physicians’ anesthesiology, neurology, and neurosurgeons, who signed off on the brain death report. The demographic information of the participants was recorded. A total of 31 questions in the questionnaire were about personal experiences, attitudes toward brain death, organ donation, and donor care, and the level of knowledge and expertise required for the identification and care of potential organ donors. The answers are "agree," "indecisive," and "disagree."

Results

A total of 29 physicians, seven (24.1%) neurologists, six (20.7%) neurosurgeons, and 16 (55.2%) anesthesiologists answered the questionnaire. Although all of the participants stated that brain death is a definite death, it was determined that they did not agree on how the process should proceed for non-donors after the diagnosis of brain death.

Conclusion

Physicians' attitudes towards deceased organ transplantation are positive. It is pointed out that society's insensitivity and indifference to the decrease in organ donation rates. Multidisciplinary work motivation may increase deceased organ transplant rates.

## Introduction

Increasing deceased organ transplantation is essential for countries to overcome organ shortages. Two crucial steps in the process of organ transplantation involving deceased donors are the determination of brain death and the possibility of the family refusing to donate the organs. For nations with a low rate of organ transplantation from living donors, the provision of organs from the deceased is a strategy for addressing organ shortages. The organ donation approach is based on teamwork, mutual understanding, and socially defined practice [1]. Organs are valuable not only to nations but also to zones within nations. Particularly in our region of Northern Cyprus, consistent and decisive research should be conducted to enhance the number of deceased organ transplants for patients without a living donor. Due to its limited population for organ procurement and lack of international sharing, the availability of organs is restricted. Although there is a protocol on organ sharing and supply between Turkey and Northern Cyprus.

In Northern Cyprus, in 2016, first living and then deceased organ transplantation began under the auspices of Hacettepe University. During this period, organs were shared with Turkey for heart, liver, and corneal transplants, which were not performed in Northern Cyprus. Later, heart transplants started to be performed in Northern Cyprus, again from Turkey, under the auspices of Akdeniz University. However, we cannot say that any organ transplant or sharing has been done from Turkey to Northern Cyprus since the beginning of the protocol. Despite this, such a protocol exists between Turkey and Northern Cyprus and is still in effect. For this reason, Northern Cyprus will continue to do its own work to increase deceased organ transplantation and, at the same time, maintain international cooperation.

In 2016, the first deceased organ transplant was conducted in Northern Cyprus. Prior to the COVID-19 pandemic, the rate of organ transplants from deceased donors was greater; however, the rate of transplants from living donors increased after the epidemic. Nalbantoğlu State Hospital is the only state hospital in Northern Cyprus that provides tertiary health care. It is also the largest hospital in the country, with a capacity of 500 beds. Unfortunately, for a long time during the pandemic, this hospital was transformed into a pandemic hospital, and the COVID (-) patients were directed to other public and private hospitals with an arrangement. In this hospital, which has the only organ transplant certificate in the country, the rates of living and deceased organ transplants decreased during the pandemic. Transplant surgery is performed in this hospital without transferring or sharing two kidney units obtained from each deceased person with another hospital. Due to this advantage, before the pandemic, the number of deceased organ transplant patients was higher than the number of living ones. However, after the pandemic, the number of living donor transplant patients, who were probably waiting and postponing, increased, and the rate became equal. Understanding the challenges of getting organs from living donors, delving into the perspectives, awareness, and experiences of physicians involved in diagnosing brain death, as well as examining the attitudes of various segments of society, can yield valuable insights to boost deceased organ transplantation [2].

The legal diagnosis of brain death in Northern Cyprus requires the input of an anesthesiologist, a neurosurgeon, and a neurologist, each from a different department, to concur on the report. The diagnosis of brain death must also be supported by brain computed tomography angiography in addition to brain stem reflexes. In Northern Cyprus, a diagnosis of brain death is essential for deceased organ transplantation. Being a donor for the health of the deceased person and having a document confirming this situation is only a request. After brain death, the family makes the final decision to become a donor.

In general opinion, it would be beneficial to analyze the attitudes and knowledge of healthcare providers involved in the diagnosis and treatment of brain death about brain death and organ transplantation in order to more efficiently manage the organ transplantation program [2,3].

In this study, we aimed to evaluate the attitudes and knowledge of anesthesiology, neurology, and neurosurgery physicians, who have signature authority in the diagnosis and reporting of brain death, towards the diagnosis of brain death and organ transplantation.

## Materials and methods

Study design

The evaluation of the attitudes and knowledge of physicians working in the diagnosis of brain death in dead organ transplantation in Northern Cyprus constitutes the main purpose of the study. This study was conducted over a two-month period (January to February 2023) through a literature review and interview method at Dr. Burhan Nalbantoglu State Hospital in Nicosia, Cyprus.

Inclusion and exclusion criteria

All participants were informed about the voluntary basis and their right to withdraw at any point. The questionnaire was completed by interviewing 34 physicians from four intensive care units. Five physicians refused to fill out the questionnaire and were excluded from the study.

Sample size and sampling technique

The study population consisted of physicians from three departments: an anesthesiologist, a neurologist, and a neurosurgeon, who signed the brain death report. The demographic information of the participants was recorded. A total of 31 questions in the questionnaire were about personal experiences, attitudes toward brain death, organ donation, and donor care, and the level of knowledge and expertise required for the identification and care of potential organ donors. The answers are "agree," "indecisive," and "disagree."

Data analysis

After collecting all the survey data, the data were analyzed descriptively using IBM SPSS Statistics for Windows, Version 24.0 (released 2016; IBM Corp., Armonk, New York, United States)

Ethical considerations

This study was conducted in accordance with the guidelines of the Declaration of Helsinki, and ethical approval was obtained from the Northern Cyprus Ministry of Health’s Ethics Committee. (Number: YTK.1.01-EK51/22)

Questionnaire protocol

The survey was conducted in the Turkish language. Questions were asked about ethics, the scientific situation, attitudes and views on the legal structure, the attitude of the hospital administration and the government, relations with other departments using intensive care, the situation of the organ transplant coordinator, and the effects on the public and the media of deceased organ transplantation in Northern Cyprus. The English version of the questionnaire and answers are provided.

## Results

A total of 29 physicians, seven (24.1%) neurologists, six (20.7%) neurosurgeons, and 16 (55.2%) anesthesiologists answered the questionnaire. The mean age of participants was 41.82±7.76. Of the respondents, 14 (48.3%) were female and 15 (51.7%) were male. All the physicians were experts in their fields. Two (6.9%) of them had less than one year of experience; five (17.2%) of them had one to three years of experience; one (3.4%) of them had three to five years of experience; and 21 (72.4%) of them had more than five years of professional experience. All of the physicians were aware of the existence of an organization assigned to detect brain death in the hospital. The number of those who did not detect brain death during their working period was seven (24.1%), the number of those who did around one to three times was 19 (65.5%), and the number of those who did >3 was three (10.3%). The number of those who know the law on the detection of brain death is 24 (82.8), and the number of those who have never read the relevant law is five (17.2%). Regarding the legislation, those who read it expressed no concerns. Only three individuals (10.3%) received training on donor procurement, while the remaining twenty-six individuals (89.7%) did not. The organ transplant coordinator's presence in the hospital was known to all participants. Nevertheless, 24 physicians considered the organ transplant coordinator competent. The remaining five physicians deemed the organ transplant coordinator to be only moderately adequate. While 15 (51.7%) participants said that the critical care physician should handle the donor process, 14 (48.3%) people stated that the donor process should be handled in conjunction with the organ transplant coordinator. To the question, "What is your personal view, endorsed by three physicians, in the event of brain death in the absence of organ donation?" Twelve (41.4%) said the patient should be detached from the respirator, eight (27.6%) said medical support should be discontinued but the patient should not be detached from the respirator until cardiac arrest occurs, and nine (31%) said treatment should not be interrupted until cardiac arrest occurs. The reasons for not detecting adequate brain death were interpreted by physicians as indifference and insensitivity by 20 (69%), intensive care unit work by five (17%), coordinator and coordination by two (6.9%), and lack of knowledge and experience by two (6.9%). From the physician's point of view against the requirement of computerized tomography brain angiography in the detection of brain death, 26 (89.7%) said that it should be applied in every case, and three (10.3%) argued that it should be left to the authority of the physician to be applied when necessary. The English version of the questionnaire and answers are shown in Table 1.

**Table 1:** Questionnaire assessing the knowledge and attitude of physicians responsible for brain death diagnosis

Questions	Answers n (%)		
	Agree	Indecisive	Not Agree
1. According to the laws, the definition of brain death confirms an absolute end of life	17(58.6)	5(17.2)	7(24.1)
2. According to scientific evidence, brain death is a definite form of death	29(100)	-	-
3. As the physician of a deceased patient, it is ethical to sign the brain death certificate	26(89.7)	3(10.3)	-
4. As the physician of the deceased patient, it is legally permissible to sign the brain death certificate	23(79.3)	2(6.9)	4(13.8)
5. Brain death detection should be compulsory in intensive care units	27(93.1)	2(6.9)	-
6. Problems are experienced with other responsible consultant physicians while detecting brain death	5(17.2)	4(13.8)	20(69)
7. Studies of the national coordination system between brain death detection and donation and organ donation are sufficient	16(55.2)	8(27.6)	5(17.2)
8. Brain death detection process and donor care significantly disrupt intensive care work	3(10.3)	1(3.4)	25(86.2)
9. I do not find the attitude of the coordinators towards intensive care units correct	3(10.3)	7(24.1)	19(65.5)
10. Intensive care personnel react while detecting brain death	2(6.9)	27(93.1)	-
11. The view of the administrative structure towards the donor organization in our hospital is quite positive	20(69)	8(27.6)	1(3.4)
12. The supply of cadaver donors is one of the parameters that determine the quality of hospitals and intensive care units	24(82.8)	2(6.9)	3(10.3)
13. The supply of cadaver donors makes hospitals and intensive care units lose prestige	1(3.4)	2(6.9)	26(89.7)
14. Donor procurement should have economic benefits for the intensive care physician	-	5(17.2)	24(82.8)
15. Brain death detection causes intensive care to have problems with media	5(17.2)	9(31)	15(51.7)
16. Brain death detection causes intensive care to have problems with the courthouse	1(3.4)	8(27.6)	20(69)
17. I have sufficient knowledge about donor care	16(55.2)	11(37.9)	2(6.9)
18. I have sufficient knowledge and experience in brain death detection	29(100)	-	-
19. Brain death detection causes the intensive care unit to have problems with other units	1(3.4)	8(27.6)	20(69)
20. The transplantation center in TRNC is scientifically quite sufficient	4(13.8)	15(51.7)	10(34.5)
21. I think the distribution of cadaveric organs within the national system is completely fair	10(34.5)	17(58.6)	2(6.9)
22. Transplant teams who come for organ harvesting cause problems in the hospital operation	7(24.1)	12(41.4)	10(34.5)
23. The transplantation center in TRNC is sufficient for the implementation of ethical rules	12(41.4)	14(48.3)	3(10.3)
24. I think the organ transplant coordinator is very important in supplying cadaver organs	29(100)	-	-
25. The hospital administration and the Ministry of Health support the system sufficiently	5(17.2)	18(62.1)	6(20.7)
26. Intensive care is given sufficient importance by the state in the transplantation system of the country	7(24.1)	10(34.5)	12(41.4)
27. Transplantation centers are aware of the importance of intensive care	11(37.9)	15(51.7)	3(10.3)
28. Brain death declaration should be made by the intensive care physician to the family	7(24.1)	7(24.1)	15(51.7)
29. The request for organ donation from the family can be made by the intensive care physician	4(13.8)	7(24.1)	18(62.1)
30. In my opinion, I do not believe that the notion of a potential donor with a GKS score lower than 5 is necessary or ethical	11(37.9)	4(13.8)	14(48.3)
31. It is unnecessary to report patients who are unfit for donation to the system	12(41.4)	13(44.8)	4(13.8)

## Discussion

The purpose of this study is to investigate one of the primary stakeholders to improve the rates of deceased organ transplants. In the study, it was tried to evaluate the attitudes and knowledge of the physicians from three departments (neurology, anesthesiology, and neurosurgery) who are in the diagnosis of brain death and have legally signed the brain death document. This study was conducted at the only transplant center in the region, which is a limitation of the research. However, the fact that the study does not only agree with the anesthesiologist but also evaluates other departments that contribute and sign the diagnosis reveals its privilege in terms of integrity. The study group consists of physicians from three different departments without subgroups, and the small number of participants constitutes the other limitation of the study. Moreover, this study did not involve other stakeholders in the multidisciplinary study of deceased organ transplantation, such as radiologists, other physicians and nurses, and the rest of the health workers. In terms of multidisciplinary work and motivation, thoughts only represent the thoughts and attitudes of physicians who are authorized to sign the diagnosis of brain death. This adds another limiting factor to the research.

The fact that 5% of the participants in the study on deceased organ transplantation, which is a legally regulated procedure, have never read the relevant law is contradictory and creates a concern for these physicians. In addition, only 10.3% of the participants were trained in the diagnosis and care of deceased donors. The number of physicians who did not diagnose brain death until the completion of the study was 24.1%. Physicians' perceptions of organ donation can be positively influenced by educating them about end-of-life care and the organ donation process. This can increase the potential for organ donation [4,5].

Almost half of the participants argued that the anesthesiologist should undertake the donor process in the intensive care unit. However, the majority of the physicians participating in the study thought that the coordinator had sufficient competence in this process. A trained brain death coordinator can improve communication with donors' families and enhance the quality of the process [5].

In the case of brain death without organ donation, the lack of consensus on how the process should be continued shows that there is a contradiction and doubt about the diagnosis of brain death. Likewise, the thought that a group of 10.3% of physicians should leave brain angiography to the physician's authority is related to the lack of agreement between departments in the diagnosis of brain death. In a study conducted in Turkey, the rate of those who stated the necessity of using supportive tests evaluating cerebral blood flow was 87.5%, while this rate was calculated as 89.7% in our study [6].

In the study conducted in Turkey, approximately 69% of the participants agreed that life support should be discontinued for people who were diagnosed with brain death but whose organ donation was rejected. However, 41.9% of the participants state that circulation should be taken into account in the decision to terminate life support [6]. According to a study conducted in Pakistan, 31% of the participants deemed it inappropriate to withdraw respiratory support following brain death [7]. According to our study, the majority (58.6%) believe that respiratory support should continue even after brain death, as long as there is still circulation and a heartbeat present. These findings show that although physicians discursively and scientifically accept brain death as a definite death state, they are skeptical of brain death when it is exemplified or experienced in real life.

The rates of deceased kidney transplantation and living donor kidney transplantation are comparable in Northern Cyprus. According to the territory in which we reside, the success of deceased kidney transplantation rates demonstrates the significance of the expertise and positive attitude of physicians. In a study conducted in Iran, it was emphasized that having a multidisciplinary team that believes in brain death and organ transplantation will increase the number of organ transplants [1]. In general, physicians do not have concerns about the definition of brain death or the ethical and legal situation surrounding its diagnosis. There are no economic prospects for physicians who report a diagnosis of brain death. Participants who did not indicate a lack of knowledge and expertise in the diagnosis of brain death did not hold the same attitude regarding their knowledge and experience in donor care. In addition, they are aware of the fact that the diagnosis of brain death and deceased organ transplantation increases the prestige and quality of intensive care units and hospitals. This positive feedback is encouraging for the diagnosis of brain death and the transplantation of deceased organs. However, the disruptions in the organization of the brain death diagnosis process, the lack of government support, and the indecision and polarization of physicians about multidisciplinary collaboration are the remarkable points.

## Conclusions

According to our study, even if the attitude of physicians is positive, establishing well-structured education programs, emphasizing state aid and support, and raising public awareness to eliminate the indifference and insensitivity of the public can increase the number of deceased organ transplants. Motivation and encouragement of multidisciplinary work may increase the number of deceased organ transplants.
